# Associations between Malocclusion and Oral Health-Related Quality of Life among Mongolian Adolescents

**DOI:** 10.3390/ijerph14080902

**Published:** 2017-08-10

**Authors:** Miyu Araki, Yuko Yasuda, Takuya Ogawa, Tsasan Tumurkhuu, Ganjargal Ganburged, Amarsaikhan Bazar, Takeo Fujiwara, Keiji Moriyama

**Affiliations:** 1 Department of Maxillofacial Reconstruction and Function, Division of Maxillofacial/Neck Reconstruction, Graduate School of Medical and Dental Sciences, Tokyo Medical and Dental University, Tokyo 113-8510, Japan; miyumort@tmd.ac.jp (M.A.); y-yasuda.mort@tmd.ac.jp (Y.Y.); t-ogawa.mort@tmd.ac.jp (T.O.); tsasant@gmail.com (T.T.); 2 Department of Prosthodontics and Orthodontics, School of Dentistry, Mongolian National University of Medical Sciences, Ulaanbaatar 14210, Mongolia; ganjargal@mnums.edu.mn (G.G.); amarsaikhan@mnums.edu.mn (A.B.); 3 Department of Global Health Promotion, Tokyo Medical and Dental University, Tokyo 113-8510, Japan

**Keywords:** child perceptions questionnaire, malocclusion, Mongolia, oral health-related quality of life

## Abstract

Malocclusion may affect oral health-related quality of life (OHR-QoL), however, the previously detected associations were affected by confounding factors. We hypothesized that there is indeed an association between OHR-QoL and malocclusion and investigated in a population-based study of 420 Mongolian adolescents mean age: 12.6 (standard deviation (SD) = 1.09) years from two secondary schools, located in an urban and a suburban area. We randomly selected two classes from each school. The Index of Orthodontic Treatment Need (IOTN) was used to assess malocclusion. OHR-QoL was assessed using the Child Perceptions Questionnaire (CPQ). Multivariate analysis was used to determine whether malocclusion had an independent effect on OHR-QoL. Overall, the existence of any type of malocclusion was not significantly associated with CPQ results. However, increased overjet was significantly associated with oral symptoms (coefficient: 0.66, 95% CI: 0.14–1.19), functional limitations (coefficient: 0.62, 95% CI: 0.17–1.08), and social well-being (coefficient: 0.50, 95% CI: 0.06–0.93). Deep bite was also significantly associated with oral symptoms (coefficient: 0.54, 95% CI: 0.23–0.84) and functional limitations (coefficient: 0.45, 95% CI: 0.19–0.72). Although malocclusion per se was not significantly associated with OHR-QoL, specific types of malocclusion, i.e., increased overjet and deep bite, were associated with OHR-QoL.

## 1. Introduction

Oral health in children has received much attention recently, because compromised oral health influences a child’s daily life, as it is associated with oral pain, difficulty in chewing, avoidance of smiling, being teased, and skipping school [1], which in turn can affect the child’s academic performance [2]. Oral health involves not only oral diseases, but also functional aspects (e.g., chewing ability), psychological aspects (e.g., anxiety about the mouth), and social aspects (e.g., smiling or laughing) [1,2]. Therefore, oral health is a component of quality of life (QoL), and can be assessed via a series of parameters that collectively make up oral health-related quality of life (OHR-QoL) [3]. OHR-QoL is defined as the functional and psychosocial outcomes of oral disorders and its measurement covers four aspects: (1) functional factors; (2) mastication, utterance, and psychological factors; (3) esthetic, social, and communication-related factors; and (4) pain and discomfort-related factors; these factors show good validity and reliability [4,5]. OHR-QoL is assessed by using questionnaires. One of these questionnaires is the Child Perception Questionnaire (CPQ), which is a generic OHR-QoL measurement tool for use in children aged 11–14 years; the CPQ has been validated and was shown to have excellent reliability (Cronbach’s alpha was 0.89) [4].

Factors that can influence OHR-QoL include dental caries [6], gingivitis [7], and malocclusion [1]. Malocclusion may have a stronger and longer-lasting impact on OHR-QoL than these other factors, because it is associated with poor speech capability and poor chewing capability [8]. Furthermore, children with severe malocclusion can be teased at school due to their dental appearance, which influences social aspects of OHR-QoL [1,9]. In order to diagnose malocclusion, four commonly used American and European orthodontic treatment-need indices are typically used. These are the Index of Orthodontic Treatment Need (IOTN), Dental Aesthetic Index (DAI), Handicapping Labio-lingual Deviation index, and the Index of Complexity, Outcome, and Need (ICON) [10]. Among these, only the IOTN can diagnose the type of malocclusion, such as increased overjet, reverse overjet, deep bite, open bite, and crowding which we wanted to investigate. So, we selected IOTN.

It is considered that age, sex [11], dental-related issues such as tooth brushing [12] and dental caries [13], and markers of socio-economic status, such as family income [14], are associated with both malocclusion and OHR-QoL. In support of these assertions, previous studies have reported associations between malocclusion and OHR-QoL in several countries, including Brazil [15,16,17,18], Canada [11], Saudi Arabia [19], New Zealand [1], and Korea [9]. Barbosa et al. [15] reported a significant association between malocclusion and OHR-QoL in a hospital-based study in Brazil, as did Agou et al. [11] in Canada, Dawoodbhoy et al. [19] in Saudi Arabia, Foster Page et al. [1] in New Zealand, and Choi et al. [9] in Korea. In population-based studies, both Sardenberg et al. [17] and Silva et al. [18] reported a significant association between malocclusion and OHR-QoL in Brazil. Conversely, there were no significant association between malocclusion and OHR-QoL in a hospital-based study conducted by Locker et al. [20] in Canada, or in another population-based study conducted in Brazil [16]. These inconsistent findings may be due to differences in the countries involved, the age of the group studied, and the study design, i.e., whether the study was population-based or hospital-based. Thus, associations between malocclusion and OHR-QoL need to be assessed in individual countries, as results from one country cannot necessarily be extrapolated to another. However, few relevant studies have been conducted in Asian countries other than Korea [9], and the Mongoloid population has rarely been studied.

The Mongolian People’s Republic was renamed “Mongolia” in 1992, signifying a departure from socialism. This marked change in both the social and economic environments has led to lifestyle changes, including altered dietary habits. Mongolians traditionally consumed milk and meat from sheep, cows, horses, or camels, but recently have begun to incorporate sugar candies and sweetened juice into their diet, which has increased the prevalence of dental caries in Mongolia [21]. Because caries is one of the important determinants of malocclusion [13], and orthodontic treatment is developing in Mongolia, investigation of the associations between malocclusion and OHR-QoL in Mongolians is needed. 

Thus, in this study, we investigated the association between malocclusion and OHR-QoL in Mongolian adolescents, and investigated specifically whether different types of malocclusion had differential effects on OHR-QoL.

## 2. Materials and Methods

### 2.1. Samples

Samples were recruited at Ulaanbaatar, the capital city of Mongolia, in which almost half (45.8%) of the country’s total population reside, and where more than one-third of children go to school. Ulaanbaatar has nine large districts, and the two largest of these are Bayanzurkh and Songino Khairkhan. In the present study, we chose the biggest secondary schools in each of these districts, in an effort to increase the number of study participants. One school was located in the urban area of Bayanzurkh, and the other was located in the suburban area of Songino Khairkhan, to assess individuals from different backgrounds. At each school, we randomly selected two classes from each of the 6th to 9th grades (age 10–16 years, mean age 12.6 years). These adolescents are at the stage of permanent teeth eruption. We distributed informed consent forms to parents of participants. The exclusion criteria were: refusal to participate in the study (*n* = 4), absence on the day of the investigation (*n* = 77), incomplete questionnaire (*n* = 13), and previous orthodontic treatment (*n* = 29). The final sample consisted of 420 participants. The study was approved by the Ethical Review Board of the Mongolian National University of Medical Science (No. 13-12/1A) and Tokyo Medical and Dental University (No. 961).

### 2.2. Assessment of Malocclusion

While seated in a chair, participants were examined by an orthodontist with at least 2 years of orthodontic training at the Department of Maxillofacial Orthognathics, Tokyo Medical and Dental University. Posterior and anterior crossbite, lingual crossbite, upper and lower crowding, cleft of the lip and/or palate, and hypodontia were investigated by means of a dental mirror. Overjet, overbite, and the size of the upper left central incisor were measured using periodontal probes (YDM Ltd., Tokyo, Japan). This examination generally followed the method reported by Komazaki et al. [22]. We used orthopantomographic images to re-evaluate hypodontia. Malocclusion was diagnosed using the IOTN [23]. The IOTN has two components, i.e., a dental health component (DHC) and an esthetic component (AC). In this study, we used only the DHC. We defined “need for orthodontic treatment” as an IOTN grade of 4 or 5, and “no need for orthodontic treatment” as an IOTN grade of 1–3 [24]. We also diagnosed the type of malocclusion. Increased overjet was defined as an overjet of >6 mm, and reverse overjet was defined as <−1 mm. Deep bite was diagnosed where there was an overbite of >3.5 mm and impression on the palate was observed, and open bite was defined as an overbite of ≤−4 mm. If crossbite was observed, it was recorded as either anterior crossbite or posterior crossbite. If a scissor-bite was observed, it was recorded as lingual crossbite. Crowding was recorded if it was ≥5 mm.

### 2.3. Measurement of Oral Health-Related Quality of Life

We used the CPQ_11–14_ [25] to measure the impact of malocclusion on OHR-QoL. As the CPQ was originally written in English, we had it translated into Mongolian, with back translation. The instrument is made up of 35 items distributed among four domains: oral symptoms (five items), such as “bad breath”, functional limitations (nine items), such as “unclear speech”, emotional well-being (10 items), such as “nervous or afraid” and social well-being (11 items), such as “being teased” Each question began with the words “During the last 3 months, how often have you had or been…” [25]. Response options and their corresponding scores were: “Never” (scoring 0); “Once or twice” (scoring 1); “Sometimes” (scoring 2); “Often” (scoring 3); and “Every day or almost every day” (scoring 4). The total scores and the individual scores for each of the four domains were calculated (the possible range is 0–140, 0–20, 0–36, 0–40 and 0–44, respectively), with higher scores denoting a lower OHR-QoL.

### 2.4. Statistical Analysis

Demographic variables for those participants with and those without malocclusion were compared using chi-square analysis. The association between malocclusion and the total scores of CPQ was compared using *t*-test analysis. The associations between malocclusion and the total CPQ score and that of each of its four individual domains were investigated using a multivariate adjusted model, that is, a model adjusted for age, sex, family income, dental caries, and frequency of tooth brushing [11,16,19,26,27]. We categorized the family income level based on the following distribution: low, less than 500 thousand tugrik, intermediate, 51 to 100 thousand tugrik, high, more than 101 thousand tugrik. Because the prevalence of dental caries was very high (95.7%), we set a cut-off at more than five teeth, to prevent deviation. Frequency of tooth brushing was based on the study by Wakaguri et al. [12]. Stata 13 SE (StataCorp LP, College Station, TX, USA) was used for all statistical analysis. 

## 3. Results

Table 1 shows the distributions of demographic characteristics, dental status, family income level, and school location, stratified by malocclusion. The prevalence of malocclusion needing orthodontic treatment was 31.2% (95% confidence interval 26.7–35.6%). Dental caries was present in more than 5 teeth in 216 (58.2%) of the participants. With regard to family income level, 18.3% of the participants were from high income families, 51.7% were from intermediate income families, and 23.8% were from low income families. None of the covariates were significantly associated with malocclusion, as determined via the chi-square test.

Table 2 shows the distributions of malocclusion types. Increased overjet, deep bite, anterior crossbite, posterior crossbite, and crowding were present to varying degrees (2.4%, 5.5%, 4.0%, 3.8%, and 11.9%, respectively).

Table 3 shows the association between malocclusion and total CPQ score, as well as its four domains. In the no need for orthodontic treatment group, the mean score and SD of each of the four individual domains (i.e., oral symptoms, functional limitations, emotional well-being, and social well-being) were 3.83 (SD = 3.47), 5.20 (SD = 5.08), 10.76 (SD = 7.57), and 7.40 (SD = 6.19), respectively. In the need for orthodontic treatment group, these scores were 4.25 (SD = 3.48), 5.39 (SD = 5.83), 10.98 (SD = 7.15), and 7.44 (SD = 6.55), respectively, and did not differ significantly from the No need for orthodontic treatment group (all *p* > 0.2). Those who needed orthodontic treatment tended to exhibit higher total scores as well as higher scores in the four individual domains, but these differences were not statistically significant as determined by *t*-test analysis.

Table 4 shows the associations between different types of malocclusion and the four CPQ domains.

Increased overjet was significantly associated with oral symptoms (coefficient: 0.66, 95% CI: 0.14–1.19), functional limitations (coefficient: 0.62, 95% CI: 0.17–1.08), and social well-being (coefficient: 0.50, 95% CI: 0.06–0.93). Deep bite was significantly associated with oral symptoms (coefficient: 0.54, 95% CI: 0.23–0.84) and functional limitations (coefficient: 0.45, 95% CI: 0.19–0.72). Anterior crossbite, posterior crossbite, and crowding were not significantly associated with any of the four CPQ domains.

## 4. Discussion

In the current study, there was no significant association between malocclusion and OHR-QoL in Mongolian adolescents after adjustment for age, sex, family income, dental caries, and frequency of tooth brushing. However, increased overjet was significantly associated with oral symptoms, functional limitations, and social well-being. Furthermore, deep bite was significantly associated with oral symptoms and functional limitations. To the best of our knowledge, associations between specific types of malocclusion and OHR-QoL among adolescent in a population-based investigation have not been reported previously. 

This study adds to the literature, as previous studies have tended to be hospital-based [1,9,11,15,19] rather than population-based [16,18], and have yielded inconsistent results. O’Brien et al. [28] showed that malocclusion significantly affected OHR-QoL in a hospital-based study conducted in the United Kingdom on 147 children aged 11–14 years. Dawoodbhoy et al. [19] reported a significant association between malocclusion and all four CPQ domains in a hospital-based study of 278 children aged 11–14 years. Notably, the fact that these studies were hospital-based may have influenced the results, because participants who visit the hospital may tend to be more worried about malocclusion [29]. In a population-based study in Brazil, Barbosa et al. [16] reported that there was no statistically significant association between the mean CPQ score and malocclusion, which is consistent with the findings of the current study. That study used the Dental Aesthetic Index (DAI) to diagnose malocclusion; thus, different types of malocclusion were not considered. 

Because there are many different types of malocclusion, patients are likely to have different experiences in terms of appearance and function. Therefore, the associated impact on OHR-QoL is likely to differ according to the type of malocclusion present. One previous study in Brazil investigated associations between different types of malocclusion and OHR-QoL, and found that anterior segment spacing and anterior crossbite were significantly associated with OHR-QoL [17]. In contrast to this previous study, we found that increased overjet and deep bite was associated with OHR-QoL among adolescent in Mongolia. The difference may be due to the difference in the ages of the populations studied: the previous study in Brazil investigated young children, aged 8–10 years, while our sample of Mongolian children involved participants aged 10–16 years. Perception of social recognition may also differ according to age and culture. Further replication studies in other settings, using population-based adolescent samples, are needed.

The mechanism related to how a specific type of malocclusion is associated with OHR-QoL may involve the following. In patients with increased overjet, some of the highest scores derived from the question items relating to oral symptoms involving “bad breath” and “bleeding gums”, and may be because patients with increased overjet are more likely to suffer from periodontal disease [30,31]. Moreover, a habit of mouth breathing is reportedly associated with increased overjet [32], and this may lead to dry mouth, which may in turn lead to bad breath [33]. In patients with deep bite, one of the highest scores derived from the question items relating to oral symptoms on “mouth sores”. The criteria for deep bite included an impression on the palate, which can cause pain in the mouth [34]. In terms of functional limitations, some of the highest scores derived from the question items relating to “trouble chewing tough food” and “breathing through your mouth”. It is difficult for patients with increased overjet and deep bite to bite off food with their anterior teeth. The association between mouth breathing and increased overjet may also explain the associations between increased overjet, deep bite, and functional limitations [32]. Increased overjet reportedly also affects social well-being, because the associated facial configuration affects personal relationships [35,36].

In terms of prevalence of malocclusion requiring treatment, Borzabadi-Farahani et al. showed that the prevalence of malocclusion needing orthodontic treatment, as based on the IOTN, was 36.1%, and the highest prevalence of this type of malocclusion involved crowding, according to a population-based study in Iran [37,38]. Similarly, Komazaki et al. in a population-based study in Japan [22] showed that the prevalence of malocclusion needing orthodontic treatment, as based on the IOTN, was 44.9% and the highest prevalence was related to crowding. In the current study, the prevalence of such malocclusion was 31.2%, and the highest prevalence also involved crowding; thus, the results of the present study were similar to those previously reported. 

The current study had several limitations. First, we did not assess the participants’ own perceptions of their occlusion. Locker et al. [20] reported a significant association between children’s feelings about the appearance of their teeth and CPQ scores. Further studies incorporating each participant’s own subjective assessment of their malocclusion are needed. Second, a sampling bias was present, as we chose participants from only two public schools. Third, the total sample size in this study was limited to 420 individuals. Fourth, other confounding factors, for example oral habits, such as lip biting, nail biting, and bruxism, may have been present. However, oral habits vary widely, and thus it may not have been possible to effectively adjust for such habits. Fifth, malocclusion diagnosis using IOTN was defined by the single worst occlusal trait [10]; therefore, if there are two or more occlusal anomalies, only the worst trait is considered [10].

Despite these limitations, the current study had several strengths. First, we used a representative sample; thus, we can generalize the evidence to the population of Ulaanbaatar. In Ulaanbaatar, 88.8% (*n* = 165,908) of school children attend a public school. Second, the sample was representative of those who are keen on their social appearance [39]. Third, we assessed the malocclusion using an international standard, i.e., the IOTN. Fourth, the study was conducted in Mongolia, where rapid development is taking place; hence, the current study may capture the impact of social change [40] on the association between malocclusion and OHR-QoL.

## 5. Conclusions

This study shows that the presence of malocclusion per se is not significantly associated with OHR-QoL. However, increased overjet was associated with oral symptoms, functional limitations, and social well-being, and deep bite was associated with oral symptoms and functional limitations. Further research is needed to investigate the causality of associations between malocclusion and OHR-QoL by assessing the effect of providing treatment for malocclusion.

## Figures and Tables

**Table 1:** Demographic characteristics of the study subject.

Characteristic	All (N = 420)	No Malocclusion (*n* = 289)	Malocclusion (*n* = 131)	*p*
	*n* (%)	*n* (%)	*n* (%)	
Sex				
male	196 (46.7)	126 (43.6)	70 (53.4)	0.061
female	224 (53.3)	163 (56.4)	61 (46.6)	
Age (years)				
10	3 (0.7)	1 (0.4)	2 (1.5)	0.081
11	82 (19.5)	52 (18.0)	30 (22.9)	
12	96 (22.9)	64 (22.2)	32 (24.4)	
13	142 (33.8)	110 (38.0)	32 (24.4)	
14	92 (21.9)	59 (20.4)	33 (25.2)	
15	4 (1.0)	3 (1.0)	1 (0.8)	
16	1 (0.2)	0 (0.0)	1 (0.8)	
Dental caries				
0–4	155 (41.8)	109 (41.5)	46 (43.2)	0.721
≥5	216 (58.2)	154 (58.6)	62 (57.4)	
Tooth brushing				
not everyday	39 (9.3)	26 (9.0)	13 (9.9)	0.48
once a day	146 (34.8)	94 (32.5)	52 (39.7)	
over twice a day	210 (50.0)	151 (52.3)	59 (45.0)	
Family income level				
low	100 (23.8)	64 (22.2)	36 (27.5)	0.457
intermediate	217 (51.7)	149 (51.6)	68 (51.9)	
high	77 (18.3)	58 (20.1)	19 (14.5)	
School location				
outside of the city center	204 (48.6)	146 (50.5)	58 (44.3)	0.236
within the city center	216 (51.4)	143 (49.5)	73 (55.7)	

*p*-value was estimated using chi-square test.

**Table 2:** Distributions of the different types of malocclusion in the study sample.

Type of Malocclusion	*n* (%)
Increased overjet	10 (2.4)
Reverse overjet	3 (0.7)
Deep bite	23 (5.5)
Open bite	0 (0.0)
Anterior crossbite	17 (4.0)
Posterior crossbite	16 (3.8)
Lingual crossbite	0 (0.0)
Crowding	50 (11.9)
Cleft lip and/or palate	1 (0.2)
Hypodontia	38 (9.0)

**Table 3:** Associations between malocclusion and scores for the Child Perceptions Questionnaire and its four individual domains.

Questionnaire Domain	All (N = 420)	No Malocclusion (*n* = 289)	Malocclusion (*n* = 131)	*p*
	Mean (SD)	Mean (SD)	Mean (SD)	
Oral symptoms	3.96 (3.47)	3.83 (3.47)	4.25 (3.48)	0.264
Functional limitations	5.26 (5.32)	5.20 (5.08)	5.39 (5.83)	0.745
Emotional well-being	10.83 (7.43)	10.76 (7.57)	10.98 (7.15)	0.783
Social well-being	7.41 (6.30)	7.40 (6.19)	7.44 (6.55)	0.948
Total score	27.46 (18.30)	27.20 (18.21)	28.03 (18.55)	0.672

*p*-value was estimated using *t*-test.

**Table 4:** Associations between malocclusion types and scores for the Child Perceptions Questionnaire and its four individual domains.

Types of Malocclusion	Oral Symptoms		Functional Limitations		Emotional Well-Being		Social Well-Being		Total	
	*β*	95% CI	*β*	95% CI	*β*	95% CI	*β*	95% CI	*β*	95% CI
Increased overjet	0.66	0.14–1.19 *	0.62	0.17–1.08 *	0.46	−0.08–1.01	0.50	0.06–0.93 *	0.54	0.15–0.93 *
Deep bite	0.54	0.23–0.84 *	0.45	0.19–0.72 *	0.19	−0.13–0.51	0.14	−0.12–0.40	0.29	0.06–0.52 *
Anterior crossbite	0.08	−0.26–0.42	−0.09	−0.38–0.21	0.063	−0.29–0.42	0.08	−0.20–0.36	0.03	−0.22–0.29
Posterior crossbite	−0.17	−0.59–0.24	0.13	−0.23–0.50	0.15	−0.29–0.59	0.20	−0.15–0.54	0.11	−0.20–0.42
Crowding	0.25	−0.01–0.52	0.16	−0.075–0.39	0.11	−0.17–0.39	0.07	−0.15–0.29	0.12	−0.07–0.33

*β*, coefficient; CI, confidence interval; * *p* < 0.05 (*p*-value was estimated using multivariate adjusted model adjusted for age, sex, family income, dental caries, and frequency of tooth brushing).
